# The sequence effect: Character position processing in Chinese words

**DOI:** 10.3389/fpsyg.2022.877627

**Published:** 2022-10-05

**Authors:** Yancui Zhang, Mengsi Wang, Jingxin Wang

**Affiliations:** ^1^Academy of Psychology and Behavior, Faculty of Psychology, Tianjin Normal University, Tianjin, China; ^2^College of Humanities, Tianjin Agricultural University, Tianjin, China

**Keywords:** character position processing, sequence effect, Chinese, words, language

## Abstract

Numerous studies indicate that letter position processing is important for word recognition; also, the position processing of external letters (especially the initial letter) is better than that of inner letters in the Roman script. Similarly, the position processing of characters is critical in Chinese word recognition. However, the position processing pattern of characters within Chinese words is still understudied. Therefore, using a single-presentation lexical decision task with 79 university students in China, we conducted two experiments with three- and four-character words to explore this issue. The results revealed clear character position processing with transposed pseudowords. Crucially, we identified a sequence effect in Chinese character position processing within words, directly supporting the hypothesis that character-based processing occurs with Chinese words. We also discussed other possibilities in Chinese character position processing.

## Introduction

In alphabetic script languages, letter position processing is a key component in the word recognition process. For example, letter position information must be accurately identified to distinguish *listen* from *silent*. Some early models assumed that there is position-specific letter processing, which means that the processing of letter position is very strict (interactive activation model, McClelland and Rumelhart, 1981; dual-route cascaded model, Coltheart et al., 2001; multiple readout model, Grainger and Jacobs, 1996; activation-verification model, Paap et al., 1982, see also Paap et al., 2000). However, many studies have shown that letter position processing is flexible, and the evidence comes from the transposed-letter effect: Transposed pseudowords (e.g., *litsen*) are more like their base words (*listen*) than pseudowords with substituted letters (e.g., *lidfen*; Perea and Lupker, 2003, 2004; Meade et al., 2021). Transposed pseudowords have the same letters as base words, but the letter position is different. Thus, transposed pseudowords reflect letter position processing, while the difference between base words and pseudowords with substituted letters is only a matter of letter identity. As such, pseudowords with substituted letters reflect letter identity processing. Therefore, the essence of the transposed-letter effect is—leaving aside some flexibility in letter identity processing (see Marcet and Perea, 2017)—letter position processing is subject to a large degree of flexibility (the overlap model, Gomez et al., 2008; SERIOL model, Whitney, 2001; SOLAR model, Davis, 2010a; the dual-route approach, Grainger and Ziegler, 2011, see also Grainger, 2018; OB1-reader, Snell et al., 2018). If we examine letter position processing only, we can use the comparison between transposed pseudowords and base words so that letter position processing can be independent of letter identity processing (Kezilas et al., 2017).

Although letter position processing is more flexible than letter identity processing, there is indeed letter position processing, and most of the evidence come from the comparison between transposed pseudowords and base words (Johnson and Dunne, 2012; Johnson and Eisler, 2012). Moreover, numerous studies have shown that external letters—especially the initial letter (Evett and Humphreys, 1981; Aschenbrenner et al., 2017)—have more of an advantage than inner letters in position processing (Jordan et al., 2003; Guérard et al., 2012). For example, in priming studies, response times were shorter when the participants were primed with external letters of a word rather than the inner letters (McCusker et al., 1981). In letter detection tasks, the response to external letters of transposed pseudowords (especially involving the initial letter) was faster and more accurate than the response to the inner letters of the transposed pseudowords (Guérard et al., 2012). Similarly, in studies on sentence reading in Latin script with transposed pseudowords, the reading process was more significantly disrupted when letters were externally transposed, especially initial letters (White et al., 2008; Johnson and Eisler, 2012).

Why do external letters, especially the initial letter, have more of an advantage than inner letters in position processing? Although some researchers suggest that letters within words are processed serially from left to right rather than in parallel, at least for early word processing recognition (White et al., 2008), scholars generally affirm that letters within words are processed in parallel (Adelman, 2011; Grainger et al., 2016), at least for relatively short words. On this basis, letter position processing within words indicates this pattern. At present, there are six theoretical accounts to explain letter position coding during lexical processing, including slot coding, Wickelcoding, both-edges coding, noisy slot-based coding, open-bigram coding, and spatial coding. In slot coding and Wickelcoding, letter position processing is very strict (McClelland and Rumelhart, 1981; Seidenberg and McClelland, 1989), so with these forms of coding, we cannot explain why external letters (especially the initial letter) are more important than inner letters. In both-edges coding, we encode a letter’s position relative to both the beginning and end of the word (Fischer-Baum et al., 2011). In noisy slot-based coding, each letter is activated to the maximum extent within its correct letter position, but activation also spills over to adjacent letter positions in the form of a Gaussian function (Gomez et al., 2008; Davis, 2010b; Norris and Kinoshita, 2012). Thus, both-edges coding and noisy slot-based coding can explain the advantage of external letters but not the special advantage of the initial letter.

As for open-bigram coding, there are two versions: discrete activation (Schoonbaert and Grainger, 2004) and continuous activation (Whitney, 2001). A letter string is coded in terms of all the ordered letter pairs it contains; for example, *calm* can activate the open-bigram as *ca*, *al*, *cm*, *al*, *am*, and *lm*. In discrete activation, the activity of the open bigram is 0 *or* 1, while in continuous activation, the activity of the open bigram is 0 *to* 1. In continuous activation, letters closer to the beginning of the word are activated earlier and to a greater extent; bigrams have higher activation levels if the letter inputs are more highly activated and if the component letters are closer together (White et al., 2008). Thus, continuous open-bigram coding can explain the advantage of external letters as well as the special advantage of initial letters. When it comes to spatial coding, all letter units are independent of position context, and letter position is coded dynamically, while the relative order of the letters in a letter string is encoded by the relative pattern of activities across letter nodes (Davis and Bowers, 2006; Davis, 2010b). As such, spatial coding can explain the advantage of initial letters but not that of end letters.

As part of orthography, letter position processing may be influenced by high-level factors and visual factors. Which kind of factor is more important? Johnson and Eisler (2012) designed four experiments to explore this. The participants read sentences containing normal words, first-two-letter transposed pseudowords, inner-two-letter transposed pseudowords, and last-two-letter transposed pseudowords. Experiments 1 and 2 also included some sentences where the spaces were removed and replaced with hash signs (#) to equate the crowding for all letters. In Experiment 3, equating was done by adding an additional space between all the letters. In Experiment 4, readers read sentences from right to left so that word-initial letters were presented furthest into the parafovea. The results showed that the advantage of initial letters was mainly related to high-level factors, while the advantage of final letter position processing was only caused by a low level of visual perception.

Interestingly, the study of Thai found that initial letter position processing was very flexible (Perea et al., 2011), and there was not only an advantage for the initial letter but also for the second letter in letter position processing for some words (Winskel et al., 2018). The pattern of letter position processing within a word was influenced by language features (Perea et al., 2018). The Chinese language uses a logographic script (Yang et al., 2020) with equal inter-character spaces and no special word spaces. Chinese characters differ from both the letters and words in other languages. They are meaningful and can either signify their own words or be combined with other characters to form two-, three-, four-, or even five-character words. The position processing of characters is vital for reading comprehension. This function enables us to distinguish 领带 (*necktie*) from 带领 (*lead*). Although many studies revealed the obvious transposed-character effect, they also confirmed the importance of Chinese character position processing, showing that the transposed-character non-words were, respectively, longer than base words with two characters or four characters in lexical decision tasks or sentence reading (Gu et al., 2015; Gu and Li, 2015; Xu and Sui, 2018; Yang et al., 2019, 2020, 2021; Chang et al., 2020). However, the position processing pattern of characters within a word is still unclear and worth exploring.

Due to the features of Chinese characters and words, whole-word recognition and character-based word recognition hypotheses have always existed in Chinese word recognition. The whole-word hypothesis states that a word is processed as a whole. Thus, each Chinese character within a word is processed in parallel and simultaneously (Li and Logan, 2008; Li et al., 2009). The character-based hypothesis posits that the character is the most fundamental component in Chinese, and the processing of a word is characterized by serial processing from left to right (Yin et al., 2011). If Chinese word recognition is based on parallel processing, and no other mechanism is formed through long-term reading in the Chinese language, the position processing of characters within words should be consistent. If Chinese word recognition is grounded in the character-based hypothesis, there would be a sequence effect of character position processing within words. Perhaps, there is another case; that is, the Chinese characters within a word are also processed in parallel, but the letter position processing within words is inconsistent. It would be interesting to see if letter position coding accounts can provide explanations.

In order to be consistent with the lower crowding of the first and last letters of English words, we used the method of lexical presentation rather than sentence reading. Meanwhile, following classic letter transposition experiments (O’Connor and Forster, 1981; see also Marcet et al., 2018), we employed a single-presentation lexical decision task. As for variable control, we used base words and transposed pseudowords, not including substituted pseudowords. As described above, this can make letter position processing independent of letter identity processing (Kezilas et al., 2017). Based on these, we conducted two experiments with three- and four-character words to determine the relative importance of the position of characters within Chinese words. In Experiment 1, we used three-character words (compound words) as basic materials to form three conditions: base words (e.g., 奥运会, *Olympic Games*, ID), first-two-character transposed pseudowords (e.g., 运奥会, F pseudowords), and last-two-character transposed pseudowords (e.g., 奥会运, L pseudowords). In Experiment 2, we used four-character words (compound words) as basic materials to form four conditions: base words (e.g., 张灯结彩, *hanging up lanterns* and *putting up decorations*, ID), first-two-character transposed pseudowords (e.g., 灯张结彩, F pseudowords), inner-two-character transposed pseudowords (e.g., 张结灯彩, I pseudowords), and last-two-character transposed pseudowords (e.g., 张灯彩结, L pseudowords). During the analysis, we compared the transposed pseudowords and base words to establish whether (Chinese) character position processing had occurred. A more important examination was the comparison between the different forms of transposed pseudowords, focusing on the position processing differences of Chinese characters within Chinese words.

## Materials and methods

The research ethics committee of the Academy of Psychology and Behavior at the author’s University approved this study, and the research was conducted in accordance with the principles of the Declaration of Helsinki.

### Participants

The participants included 79 young adults (39 in Experiment 1 and 40 in Experiment 2) aged 18–20 from Tianjin Agricultural University. All participants were undergraduate students and native Chinese readers; they all had normal vision (or vision corrected to normal), and they received 10 or 15 Yuan as compensation for their voluntary participation. The sample size was guided by the work of Xu and Sui (2018), who investigated Chinese character position processing using 70 items (3 × 5 design) with a sample size of 20. We obtained written consent from all participants prior to commencing the study.

### Stimuli and design

In Experiment 1, we chose 78 three-character words from the SUBTLEX-CH database (Cai and Brysbaert, 2010) as the basic materials to form three conditions (ID, F pseudowords, and L pseudowords). We adopted a Latin square design, in which each participant read one version of each word condition and equal numbers of words in each condition. There were three lists, each with 78 items (26 items per condition). Only one list was used for each participant. In Experiment 2, 96 four-character words were selected as the basic materials to form four conditions (ID, F pseudowords, I pseudowords, and L pseudowords). As in Experiment 1, there were four lists, each with 96 items (24 items per condition). Only one list was used for each participant. The selected base words in the two experiments met the conditions whereby every character in a word was different. Any two adjacent characters in the pseudowords could not constitute a real word, and an entire pseudoword was not a real word. Because compound words make up a major portion of modern Chinese vocabulary (Zhan et al., 2013), especially three-character and four-character words, all the words we chose were compound words. Further, in our experiments, there was no significant difference between the stroke number and frequency of the two transposed characters and those of the corresponding Chinese characters in base words (*ps* < 0.05). To make the proportion of real and false words equal, we added 26 three-character and 48 four-character real words in Experiments 1 and 2, respectively. There were 6 and 12 practice strings in Experiments 1 and 2, respectively. Therefore, Experiment 1 had 110 items per list, and Experiment 2 had 156 items per list.

### Apparatus and procedure

We used E-Prime (Version 2.0.10) to present the stimuli on a 24-inch LED screen in 32-point Song font (a normal font size for reading). A black font was presented on a white background. Each character was subtended at approximately 1° at a viewing distance of 60 cm. The participants responded using the computer’s *M* and *Z* keys on the keyboard, representing word and pseudoword decisions, respectively.

For each trial, the same sequence was followed according to the work of Marcet et al. (2018). First, a fixation point (marked by the symbol + in 32-point Song font) was presented at the center of the screen for 500 ms. Next, a target stimulus was presented and remained on the screen until the participant responded or after 2,100 ms. The participants were asked to quickly determine, as best they could, if the character string was a word by pressing the *M* key for words or the *Z* key for pseudowords, and they were instructed to keep their error rate as low as possible. The order of presentation of the stimuli changed randomly for each new participant. The experimental sessions lasted approximately 10 and 15 min for Experiments 1 and 2, respectively.

## Results

**Figure 1:**
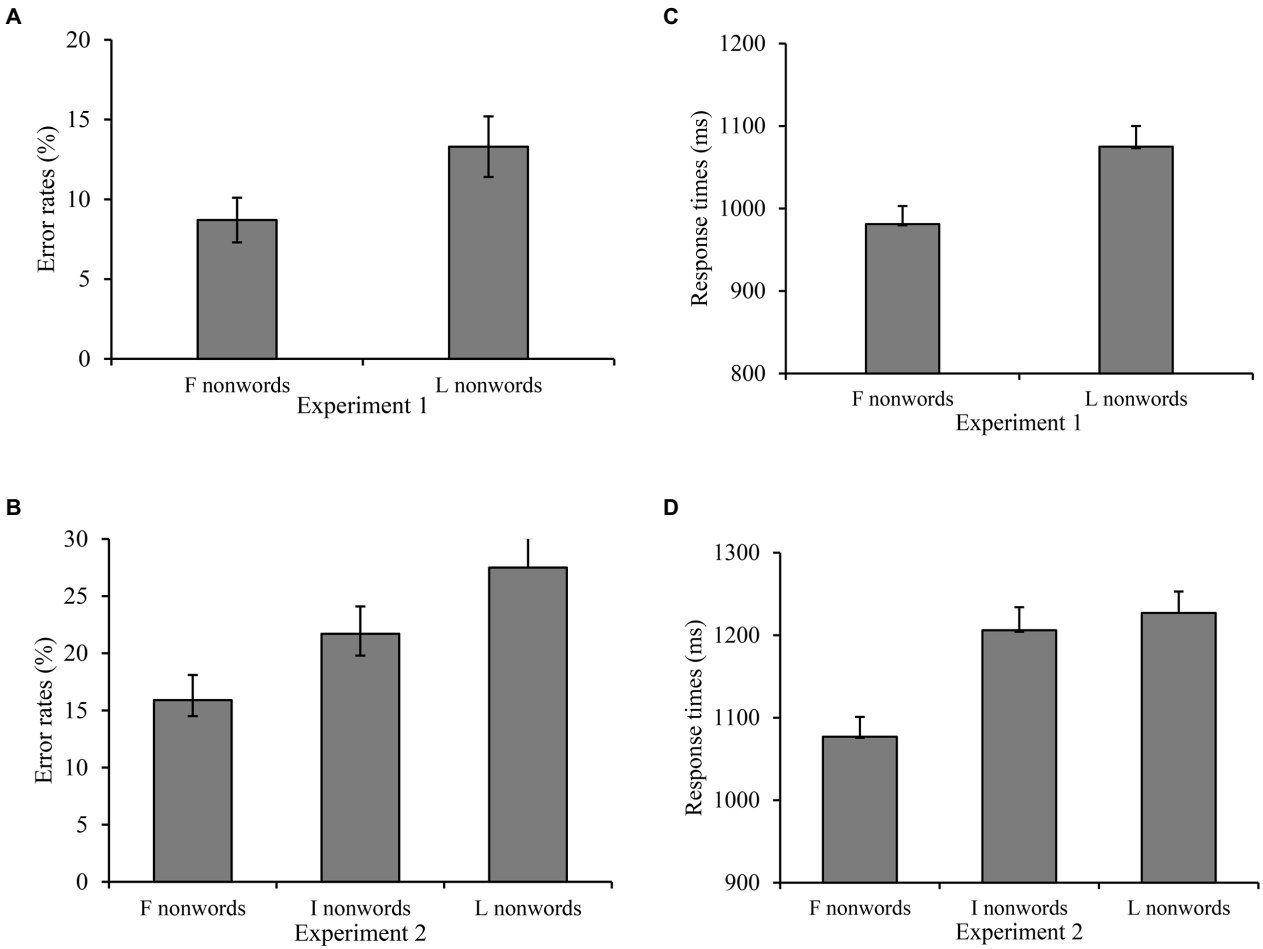
Error rates **(A,B)** and RT **(C,D)** in Experiments 1 and 2. The SE of mean is represented by the error bars. F pseudowords, first-two-character transposed pseudowords; I pseudowords, inner-two-character transposed pseudowords; and L pseudowords, last-two-character transposed pseudowords.

We excluded latencies beyond the 250–2000 ms cutoff (0.09% in Experiment 1 and 2.7% in Experiment 2). Table 1 depicts the error rates and response times (the latency of correct responses), and Table 2 portrays the statistical effects. The transposed effects are graphically represented in Figure 1. Following log-transformation, we removed all trials where response times were >2.5 SD from the participant’s grand mean.

**Table 1:** Mean error rates and response times in the three-character conditions in Experiment 1 and the four-character conditions in Experiment 2.

	Experiment 1			Experiment 2			
	ID	F pseudowords	L pseudowords	ID	F pseudowords	I pseudowords	L pseudowords
Error rate (%)	5.4 (0.8)	8.7 (1.4)	13.3 (1.9)	8.3 (1.4)	15.9 (2.2)	21.7 (2.4)	27.5 (2.6)
RTs (ms)	918 (19)	981 (22)	1,075 (25)	1,081 (27)	1,077 (24)	1,206 (28)	1,227 (26)

F pseudowords, first-two-character transposed pseudowords; I pseudowords, inner-two-character transposed pseudowords; L pseudowords, last-two-character transposed pseudowords; and RT, response time.

**Table 2:** Summary of statistical differences by type of pseudowords in Experiment 1 and Experiment 2.

	Error			RT		
	*β*	*SE*	*z*	*β*	*SE*	*t*
Experiment 1						
Intercept	−2.65	0.16	−16.91	6.87	0.02	321.68
F pseudowords—ID	0.54	0.18	3.03^*^	0.06	0.01	6.55^*^
L pseudowords—F pseudowords	0.51	0.15	3.47^*^	0.09	0.01	9.25^*^
Experiment 2						
Intercept	−1.81	0.14	−12.89	7.02	0.02	306.85
F pseudowords—ID	0.79	0.15	5.14^*^	−0.00	0.01	−0.27
I pseudowords—F pseudowords	0.44	0.13	3.50^*^	0.10	0.01	10.57^*^
L pseudowords—I pseudowords	0.37	0.11	3.21^*^	0.03	0.01	3.02^*^

* Statistically significant effects at *t*/*z* > 1.96, *p* < 0.05.

F pseudowords, first-two-character transposed pseudowords; I pseudowords, inner-two-character transposed pseudowords; L pseudowords, last-two-character transposed pseudowords; and RT, response time.

We used the lme4 package (Bates et al., 2015) in R (R Development Core Team, 2016) for data analysis. To analyze the error rate (binary variables), we employed generalized logistic mixed-effect models (GLMMs). To normalize the distributions, we calculated the natural logged transformed response times before running the linear mixed-effect models (LMMs; see Bates et al., 2015) with both stimuli and the participants as random effects. Although we intended to use a maximal random structure (Barr et al., 2013), due to convergence issues, we had to remove some slopes. We show the untransformed means in Table 1 and Figure 1 for transparency. Following convention, we deemed *t*/*z* values >1.96 to be statistically significant.

### Error rates

The results of Experiment 1 and Experiment 2 indicated that the error rate of transposed pseudowords in any form was significantly higher than that of base words (e.g., F pseudowords-base words in Experiment 1: *b* = 0.54, *SE* = 0.18, *z* = 3.03; F pseudowords-base words in Experiment 2: *b* = 0.79, *SE* = 0.15, *z* = 5.14). This implies that transposed pseudowords had additional position processing when comparing them to base words.

Crucially, in Experiment 1, the error rate of the first-two-character transposed pseudowords was significantly lower than that of the last-two-character transposed pseudowords (*b* = 0.51, *SE* = 0.15, *z* = 3.47). In Experiment 2, the error rate of the first-two-character transposed pseudowords was significantly lower than that of the inner-two-character transposed pseudowords (*b* = 0.44, *SE* = 0.13, *z* = 3.50). The error rate of the inner-two-character transposed pseudowords was significantly lower than that of the last-two-character transposed pseudowords (*b* = 0.37, *SE* = 0.11, *z* = 3.21; see Figures 1A,B).

### Response times

The outcomes of Experiment 1 demonstrated that the response time of transposed pseudowords in any form was significantly higher than that of the base words (F pseudowords-base words, *b* = 0.06, *SE* = 0.01, *t* = 6.55). The results of Experiment 2 revealed no significant difference between the first-two-character transposed pseudowords and the base words (*b* = −0.00, *SE* = 0.01, *t* = −0.27). However, the response time for the other transposed pseudowords was significantly greater than for the base words (I pseudowords-base words, *b* = 0.10, *SE* = 0.01, *t* = 10.55).

Crucially, in Experiment 1, the response time of the first-two-character transposed pseudowords was significantly shorter than that of the last-two-character transposed pseudowords (*b* = 0.09, *SE* = 0.01, *t* = 9.25). In Experiment 2, the response time of the first-two-character transposed pseudowords was significantly shorter than that of the inner-two-character transposed pseudowords (*b* = 0.10, *SE* = 0.01, *t* = 10.57). In addition, the response time of the inner-two-character transposed pseudowords was significantly shorter than that of the last-two-character transposed pseudowords (*b* = 0.03, *SE* = 0.01, *t* = 3.02; see Figures 1C,D).

## Discussion

We completed two experiments using a single-presentation lexical decision task with three- and four-character words as basic materials to explore the position processing pattern of characters within Chinese words.

By comparing transposed-character pseudowords and base words, we found that the response times to words were no faster than the transposed-character pseudowords in the initial transpositions in four-character words, while there was some advantage in three-character words. This may indicate that compared to their base words, the first-character position processing of four-character words is stricter than that of three-character words. Hence, the participants could have a shorter time to conclude that the first-two-character transposed pseudowords of four-character words are not real words, just like the time needed to deduce that four-character base words are real words. This is consistent with findings that demonstrated transposed-letter non-words in the left visual field were less likely to be mistaken for their base words (Monaghan et al., 2004; Perea and Fraga, 2006). The two initial characters of three-character words involve the fixation character and one character to the left of the fixation, while the two initial characters of four-character words entail two characters to the left of the fixation.

This is also probably related to word length and the method of word recognition (e.g., serial processing, which is supported by our study). Compared with the response time of three-character base words (918 ms), the response time of four-character base words (1,081 ms) is longer because there is an additional Chinese character. However, no matter what the first-two-character transposed pseudowords of three-character or four-character words are, they all involve the first two characters in the wrong position. Thus, compared with three-character words, the response time of the four-character base words increased even more (effect: 163 ms), but the response time of the first-two-character transposed pseudowords with four characters did not increase that much (effect: 96 ms), resulting in no differences in response times between the base words and the first-two-character transposed pseudowords in four characters. However, the error rate of the four-character base words was significantly lower than that of the first-two-character transposed pseudowords in four characters. In addition, the other results of the comparison between the transposed pseudowords and base words revealed that the transposed pseudowords had a longer response time and higher error rate than the base words. This suggests that the position processing of Chinese characters took place. This finding is consistent with prior research based on two-character words (Xu and Sui, 2018).

More importantly, two features of Chinese character position processing within words were uncovered by comparing different forms of transposed pseudowords in experiments 1 and 2. First, the superiority of initial character position processing was noticeable since the error rate and response time of the first-two-character transposed pseudowords were significantly lower and shorter than those of the last-two-character transposed pseudowords in Experiment 1, as well as that of the inner- and last-two-character transposed pseudowords. This is in line with the first letter advantage in position processing in the Roman script (Guérard et al., 2012; Aschenbrenner et al., 2017).

Second, there was a sequence effect in character position processing within words. Specifically, from left to right, the position processing of characters within words was gradually coarse and flexible. Because the position processing of characters was coarser and more flexible, the participants were more likely to confuse the transposed pseudowords with the base words, resulting in a higher error rate and longer response time. The error rate and response time of the last-two-character transposed pseudowords were higher and longer than those of the first-two-character transposed pseudowords in Experiment 1. Meanwhile, the error rate and response time of the last-two-character transposed pseudowords were higher and longer than those of the inner-two-character transposed pseudowords, and the error rate and response time of the inner-two-character transposed pseudowords were higher and longer than those of the first-two-character transposed pseudowords. Hence, unlike the letter position processing within words, there is no apparent advantage attached to final character position processing within Chinese words. In fact, the position processing of final characters was associated with a higher error rate and longer response time. Overall, the position processing pattern of Chinese characters within words is not entirely the same as letter position processing. However, there is an obvious sequence effect.

In the Roman script, there is an advantage with initial letter position processing. One reason may be that letters within words are processed serially from left to right rather than in parallel, at least for early word processing (White et al., 2008). However, scholars generally assert that the letters that comprise words are processed in parallel (Adelman, 2011; Grainger et al., 2016), at least for relatively short words. Even on this basis, there is a pattern whereby the position processing of external letters—especially the initial character—has an advantage. Johnson and Eisler (2012) found that the advantage of initial letters was mainly related to high-level factors but not to visual perception. Interestingly, even random strings that made no sense still had an advantage with the initial letter. In order to explain this, the modified receptive field (MRF) theory was proposed. According to MRF theory, with the increase in reading experience and the improvement of reading ability, the shapes of letter detectors in fixation-centered, visual receptive fields (perceptual span) become smaller and smaller for the finer processing of letters as left extension occurs (Tydgat and Grainger, 2009; Chanceaux and Grainger, 2012). Why is there an advantage in the position processing of the end letter? The results of Johnson and Eisler (2012) suggest that this is related to visual perception (e.g., less crowding). If crowding is equal for all the letters, the advantage of the end letter disappears. In fact, except for continuous open-bigram coding, no other account of letter position coding can fully explain the letter position processing pattern within words. They either explain one aspect or the other, but not both.

Chinese is a very unique language, and the recognition of Chinese words is influenced by high-level factors and visual perception. In this study, if only visual crowding and visual acuity affected Chinese character position processing, there should have been no significant difference between the first-two-character and last-two-character transposed pseudowords in Experiment 1. This is because they were the same from the perspective of crowding and visual acuity. In Experiment 2, in terms of crowding, the error rates and response times of the first-two-character and last-two-character transposed pseudowords were smaller than those of the inner-two-character transposed pseudowords. This is because the first- and last-two-character transposed pseudowords were only crowded on one side, while the inner-two-character transposed pseudowords were crowded on both sides. From the perspective of visual acuity, the error rate and response time of the inner-two-character transposed pseudowords should be less than those of the first- and last-two-character transposed pseudowords. However, we did not observe this in Experiments 1 and 2. Thus, the position processing of Chinese characters within words is likely to be influenced by other high-level factors.

As for the method of Chinese word recognition, there are two hypotheses: the whole-word recognition hypothesis and the character-based word recognition hypothesis. According to the former, the characters within words are processed in parallel and simultaneously (Li and Logan, 2008; Li et al., 2009), and if there is no other mechanism formed by long-term experience with reading Chinese and the improvement of reading ability, the position processing of characters within words should be consistent. The results of Experiments 1 and 2 do not support this hypothesis. On the contrary, they strongly support the character-based word recognition hypothesis, according to which the processing of whole words is characterized by serial processing from left to right (Yin et al., 2011). The outcomes of Experiments 1 and 2 directly support the hypothesis that Chinese word recognition is rooted in characters. Moreover, we observed a sequence effect of Chinese character position processing within words. The character itself has meaning, and the word composed of the characters has meaning, and all base words in our experiments were compound words. Thus, the amount of information in Chinese characters and the complicated structure of words made word recognition a more serial process. Our results align with the letter position pattern within words, whereby letters are processed serially (Perea et al., 2015). Perea et al. (2015) studied letter position processing in sentence reading for Braille readers, who read words letter by letter from left to right *via* finger position. They found that the reading cost of the transposed-letter conditions was linear in the tactile modality (i.e., less reading cost for final transpositions).

At the same time, it is possible for Chinese word recognition to involve parallel processing with an advantage for the first character position. According to MRF theory (Tydgat and Grainger, 2009; Chanceaux and Grainger, 2012), with the increase in reading experience and the improvement of reading ability, does the visual receptive field of Chinese reading also transform, resulting in the possibility of a smaller shape and extension to the left? This is not clear and requires further research. The interesting thing is that the spatial coding model can explain the sequence effect in Chinese character positions. The spatial coding model assumes that the relative order of the letters in a letter string is encoded by a pattern of temporary values that are dynamically assigned to these letters. For example, if we recognize the word *STOP* in a letter string, then *S* is the strongest activity, followed by *T*, *O*, *P*, presenting a series of weak activation patterns (Davis and Bowers, 2006). This is just like the character position processing within Chinese words. Hence, this may be one reason to explain the sequence effect in Chinese character position processing. However, whether this is the case needs to be tested.

Further, our results may be related to the paradigm of the single-presentation lexical decision task, at least in Chinese word recognition. Before the experiments, the participants were told what the stimulus might be, including real and false words formed by two transposed characters. Thereafter, the participants would have consciously paid attention to the position information of each character and would adopt the strategy of serial processing to gradually check them from left to right according to reading habits. They made their decisions, resulting in the sequence effect in character position processing. It is important to note that some of the effects could also have been due to the nature of the task, wherein they were presented with one stimulus at a time. In normal reading, participants have to segment words, which is not simple given that there are no perceptual cues in Chinese writing (unlike the “spaces” in Roman script). Hence, the position processing pattern of characters within Chinese words may be different. Further research is required to determine if this difference affected some results.

In conclusion, we identified a sequence effect in Chinese character position processing within words; it is not the same as in the Roman script. Moreover, it is of great significance for Chinese word comprehension and the exploration of different position processing mechanisms of characters in various languages.

## Data availability statement

The datasets presented in this study can be found in online repositories. The names of the repository/repositories and accession number(s) can be found at: https://doi.org/10.6084/m9.figshare.17304254.v1.

## Ethics statement

The studies involving human participants were reviewed and approved by the Ethics Committee of Tianjin Normal University. The patients/participants provided their written informed consent to participate in this study.

## Author contributions

All authors contributed to the design of the experiment. YZ designed the stimuli, collected the data, analyzed the data, and wrote the manuscript, with critical comments from MW and JW. All authors contributed to the article and approved the submitted version.

## Funding

This work was supported by a grant from the National Science Foundation of China to JW (81771823).

## Conflict of interest

The authors declare that the research was conducted in the absence of any commercial or financial relationships that could be construed as a potential conflict of interest.

## Publisher’s note

All claims expressed in this article are solely those of the authors and do not necessarily represent those of their affiliated organizations, or those of the publisher, the editors and the reviewers. Any product that may be evaluated in this article, or claim that may be made by its manufacturer, is not guaranteed or endorsed by the publisher.
